# *N*,*N*′-Di-*tert*-butyl-*N*′′-(2-chloro­acet­yl)phospho­ric triamide

**DOI:** 10.1107/S1600536811040773

**Published:** 2011-10-05

**Authors:** Mehrdad Pourayoubi, Behrouz Elahi, Masood Parvez

**Affiliations:** a Department of Chemistry, Ferdowsi University of Mashhad, Mashhad 91779, Iran; b Department of Chemistry, University of Calgary, 2500 University Drive NW, Calgary, Alberta, Canada T2N 1N4

## Abstract

The P atom in the title mol­ecule, C_10_H_23_ClN_3_O_2_P, has a distorted tetra­hedral coordination. In the C(O)NHP(O) unit, which has *syn*-oriented phosphoryl and N—H groups, the P—N bond of 1.703 (2) Å is longer and the O—P—N angle of 103.86 (7)° is contracted compared with the respective values in the two P(O)NHC(CH_3_)_3_ units [P—N = 1.632 (2) and 1.624 (2) Å; O—P—N = 116.80 (8) and 115.32 (8)°]. In the crystal, each mol­ecule is hydrogen bonded to two adjacent mol­ecules *via* N—H⋯O hydrogen bonds, forming a linear sequence of alternating *R*_2_^2^(8) and *R*_2_^2^(12)/*R*_2_^1^(6)-fused rings along [010]. The O atom of the carbonyl group acts as a double H-atom acceptor.

## Related literature

For compounds containing a C(O)NHP(O) skeleton and related bond lengths and angles, see: Pourayoubi *et al.* (2011 ▶). For the graph-set description of hydrogen-bond motifs, see: Bernstein *et al.* (1995 ▶). For double hydrogen-bond acceptors, see: Steiner (2002 ▶).
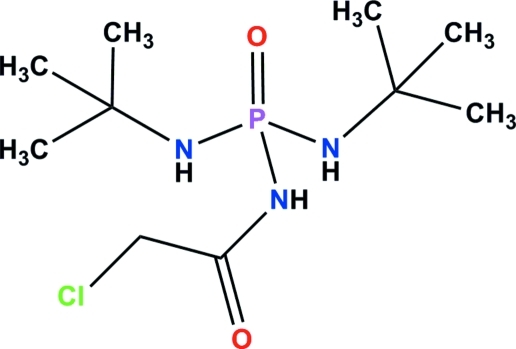


## Experimental

### 

#### Crystal data

C_10_H_23_ClN_3_O_2_P
*M* *_r_* = 283.73
Monoclinic, 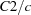

*a* = 16.4781 (5) Å
*b* = 9.8872 (2) Å
*c* = 19.6509 (6) Å
β = 111.5747 (12)°
*V* = 2977.26 (14) Å^3^
*Z* = 8
Mo *K*α radiation
μ = 0.36 mm^−1^
*T* = 173 K
0.16 × 0.14 × 0.12 mm

#### Data collection

Nonius KappaCCD diffractometer with APEXII CCD
Absorption correction: multi-scan (*SORTAV*; Blessing, 1997 ▶) *T*_min_ = 0.945, *T*_max_ = 0.958
6472 measured reflections
3410 independent reflections
2937 reflections with *I* > 2σ(*I*)
*R*_int_ = 0.026

#### Refinement

*R*[*F* ^2^ > 2σ(*F* ^2^)] = 0.043
*wR*(*F* ^2^) = 0.103
*S* = 1.10
3410 reflections
169 parameters
3 restraints
H atoms treated by a mixture of independent and constrained refinement
Δρ_max_ = 0.32 e Å^−3^
Δρ_min_ = −0.32 e Å^−3^

### 

Data collection: *COLLECT* (Hooft, 1998 ▶); cell refinement: *DENZO* (Otwinowski & Minor, 1997 ▶); data reduction: *SCALEPACK* (Otwinowski & Minor, 1997 ▶); program(s) used to solve structure: *SIR92* (Altomare *et al.*, 1993 ▶); program(s) used to refine structure: *SHELXL97* (Sheldrick, 2008 ▶); molecular graphics: *Mercury* (Macrae *et al.*, 2008 ▶); software used to prepare material for publication: *SHELXL97* and *enCIFer* (Allen *et al.*, 2004 ▶).

## Supplementary Material

Crystal structure: contains datablock(s) I, global. DOI: 10.1107/S1600536811040773/ld2026sup1.cif

Structure factors: contains datablock(s) I. DOI: 10.1107/S1600536811040773/ld2026Isup2.hkl

Additional supplementary materials:  crystallographic information; 3D view; checkCIF report

## Figures and Tables

**Table 1:** Hydrogen-bond geometry (Å, °)

*D*—H⋯*A*	*D*—H	H⋯*A*	*D*⋯*A*	*D*—H⋯*A*
N1—H1*N*⋯O2^i^	0.87 (1)	1.92 (1)	2.778 (2)	175 (2)
N2—H2*N*⋯O1^ii^	0.85 (1)	2.56 (1)	3.337 (2)	153 (2)
N3—H3*N*⋯O1^ii^	0.86 (1)	2.12 (1)	2.979 (2)	175 (2)

Symmetry codes: (i) 
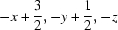
; (ii) 
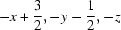
.

**Fig. 1.**
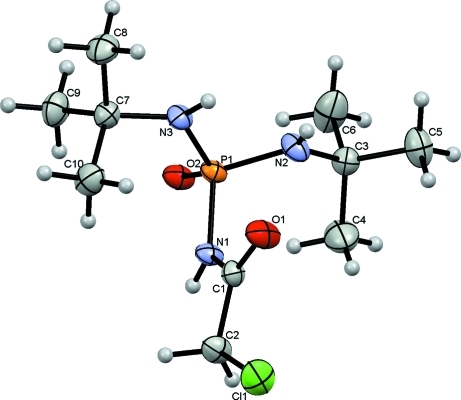
An ORTEP drawing of the title molecule with labeling. Displacement ellipsoids are given at 50% probability level.

**Fig. 2.**
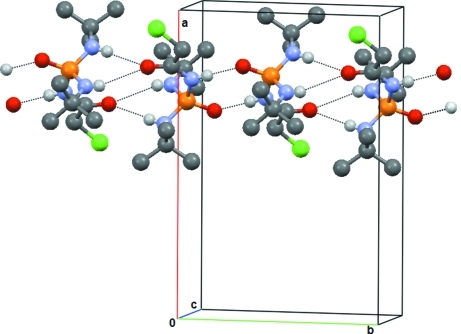
Part of the crystal packing with hydrogen bonds shown as dotted lines. Only H atoms involved in hydrogen bonds are shown.

## supplementary crystallographic information

### Comment

The structure of the title molecule, P(O)[NHC(O)CH_2_Cl][NHC(CH_3_)_3_]_2_ (Fig. 1), was determined as a part of the work on the synthesis of new phosphoramidate compounds containing a P(O)NHC(O) moiety (Pourayoubi *et al.*, 2011).

Single crystals were obtained from a solution of CHCl_3_ after slow evaporation at room temperature. The P═O and C═O bond lengths and the P—N—C bond angles are within the expected values for this category of molecules (Pourayoubi *et al.*, 2011). The P atom has a distorted tetrahedral configuration, as has been noted for the other phosphoric triamides. As expected, the P—N2 and P—N3 bonds are shorter than the P—N1 bond. In the C(O)NHP(O) moiety, the phosphoryl group has a *syn* orientation with respect to the N—H unit. The two other N—H units have an *anti* configuration relative to the P═O group.

In the crystal packing, each molecule is H-bonded to two neighbouring molecules through the N_C(O)NHP(O)_—H···(O)P hydrogen bonds and the [N—H]_2_···(O)C grouping, forming a sequence of alternated *R*_2_^2^(8) and *R*_2_^2^(12)*R*_2_^1^(6) rings (for graph-set definition in hydrogen bond motifs, see: Bernstein *et al.*, 1995) in a linear arrangement parallel to the *y* axis, Fig. 2 and Table 1. The carbonyl oxygen atom acts as a double hydrogen bond acceptor (Steiner, 2002).

### Experimental

Synthesis of CH_2_ClC(O)NHP(O)Cl_2_: The reaction of phosphorus pentachloride (33.9 mmol) and 2-chloroacetamide (33.9 mmol) in dry benzene (40 ml) at 353 K (3 h) and then the treatment of formic acid (33.9 mmol) at ice-bath temperature (2.5 h), then removing the solvent in vacuum, leads to the formation of CH_2_ClC(O)NHP(O)Cl_2_ as solid product.

Synthesis of the title molecule: To a solution of CH_2_ClC(O)NHP(O)Cl_2_ (2.42 mmol) in CHCl_3_ (20 ml), a solution of *tert*-butylamine (9.68 mmol) in CHCl_3_ (10 ml) was added dropwise at 273 K. After 4 h of stirring, the solvent was evaporated in vacuum. The obtained solid was washed with distilled water. Single crystals were obtained from a solution of the title molecule in CHCl_3_ after slow evaporation at room temperature. IR (KBr, cm^-1^): 3376, 3324, 3104, 2971, 2913, 1692, 1486, 1396, 1186, 1023, 880, 818.

### Refinement

H atoms were positioned geometrically with C—H = 0.99 and 0.98 Å for methylene and methyl H atoms, respectively, and with constrained distances N—H = 0.87 (1) Å in a riding model on their parent atoms with *U*_iso_(H) = 1.2 times *U*_eq_(N/C); the positions of H atoms of the methyl groups were rotationally optimized. Few low angle reflections were omitted as they were located behind the beam stop.

### Crystal data

**Table:**

C_10_H_23_ClN_3_O_2_P	*F*(000) = 1216
*M**_r_* = 283.73	*D*_x_ = 1.266 Mg m^−^^3^
Monoclinic, *C*2/*c*	Mo *K*α radiation, λ = 0.71073 Å
Hall symbol: -C 2yc	Cell parameters from 3520 reflections
*a* = 16.4781 (5) Å	θ = 1.0–27.5°
*b* = 9.8872 (2) Å	µ = 0.36 mm^−^^1^
*c* = 19.6509 (6) Å	*T* = 173 K
β = 111.5747 (12)°	Prism, colorless
*V* = 2977.26 (14) Å^3^	0.16 × 0.14 × 0.12 mm
*Z* = 8	

### Data collection

**Table:**

Nonius KappaCCD diffractometer with APEXII CCD	3410 independent reflections
Radiation source: fine-focus sealed tube	2937 reflections with *I* > 2σ(*I*)
graphite	*R*_int_ = 0.026
ω and φ scans	θ_max_ = 27.5°, θ_min_ = 2.5°
Absorption correction: multi-scan (*SORTAV*; Blessing, 1997)	*h* = −21→21
*T*_min_ = 0.945, *T*_max_ = 0.958	*k* = −11→12
6472 measured reflections	*l* = −25→25

### Refinement

**Table:**

Refinement on *F*^2^	Primary atom site location: structure-invariant direct methods
Least-squares matrix: full	Secondary atom site location: difference Fourier map
*R*[*F*^2^ > 2σ(*F*^2^)] = 0.043	Hydrogen site location: inferred from neighbouring sites
*wR*(*F*^2^) = 0.103	H atoms treated by a mixture of independent and constrained refinement
*S* = 1.10	*w* = 1/[σ^2^(*F*_o_^2^) + (0.0338*P*)^2^ + 4.5534*P*] where *P* = (*F*_o_^2^ + 2*F*_c_^2^)/3
3410 reflections	(Δ/σ)_max_ = 0.001
169 parameters	Δρ_max_ = 0.32 e Å^−^^3^
3 restraints	Δρ_min_ = −0.32 e Å^−^^3^

### Special details

**Table:**

Geometry. All e.s.d.'s (except the e.s.d. in the dihedral angle between two l.s. planes) are estimated using the full covariance matrix. The cell e.s.d.'s are taken into account individually in the estimation of e.s.d.'s in distances, angles and torsion angles; correlations between e.s.d.'s in cell parameters are only used when they are defined by crystal symmetry. An approximate (isotropic) treatment of cell e.s.d.'s is used for estimating e.s.d.'s involving l.s. planes.
Refinement. Refinement of *F*^2^ against ALL reflections. The weighted *R*-factor *wR* and goodness of fit *S* are based on *F*^2^, conventional *R*-factors *R* are based on *F*, with *F* set to zero for negative *F*^2^. The threshold expression of *F*^2^ > σ(*F*^2^) is used only for calculating *R*-factors(gt) *etc*. and is not relevant to the choice of reflections for refinement. *R*-factors based on *F*^2^ are statistically about twice as large as those based on *F*, and *R*- factors based on ALL data will be even larger.

### Fractional atomic coordinates and isotropic or equivalent isotropic displacement parameters (Å^2^)

**Table:**

	*x*	*y*	*z*	*U*_iso_*/*U*_eq_	
Cl1	0.94023 (4)	−0.09873 (6)	−0.09379 (3)	0.04413 (16)	
P1	0.70496 (3)	0.03761 (4)	0.02879 (3)	0.02208 (13)	
O1	0.81276 (10)	−0.15073 (13)	−0.02559 (9)	0.0381 (4)	
O2	0.67546 (9)	0.17456 (12)	0.03962 (7)	0.0287 (3)	
N1	0.77781 (10)	0.06685 (14)	−0.01251 (8)	0.0233 (3)	
H1N	0.7907 (13)	0.1494 (12)	−0.0191 (11)	0.028*	
N2	0.75351 (11)	−0.04883 (15)	0.10356 (8)	0.0284 (4)	
H2N	0.7503 (14)	−0.1342 (10)	0.0981 (12)	0.034*	
N3	0.63044 (10)	−0.06675 (15)	−0.02058 (9)	0.0248 (3)	
H3N	0.6432 (13)	−0.1496 (11)	−0.0079 (11)	0.030*	
C1	0.81982 (11)	−0.03053 (17)	−0.03486 (10)	0.0235 (4)	
C2	0.87490 (13)	0.02675 (19)	−0.07542 (11)	0.0311 (4)	
H2A	0.9129	0.0993	−0.0456	0.037*	
H2B	0.8361	0.0672	−0.1221	0.037*	
C3	0.80542 (12)	0.0073 (2)	0.17664 (10)	0.0280 (4)	
C4	0.86795 (17)	0.1160 (3)	0.17114 (14)	0.0516 (6)	
H4A	0.9015	0.1511	0.2201	0.062*	
H4B	0.8347	0.1899	0.1400	0.062*	
H4C	0.9079	0.0772	0.1498	0.062*	
C5	0.85793 (16)	−0.1082 (2)	0.22316 (12)	0.0427 (5)	
H5A	0.8914	−0.0751	0.2726	0.051*	
H5B	0.8981	−0.1440	0.2011	0.051*	
H5C	0.8182	−0.1801	0.2258	0.051*	
C6	0.74387 (16)	0.0653 (3)	0.21120 (13)	0.0521 (7)	
H6A	0.7780	0.1017	0.2597	0.063*	
H6B	0.7052	−0.0064	0.2160	0.063*	
H6C	0.7088	0.1377	0.1801	0.063*	
C7	0.56724 (12)	−0.04828 (19)	−0.09668 (10)	0.0270 (4)	
C8	0.50741 (14)	−0.1718 (2)	−0.11260 (13)	0.0392 (5)	
H8A	0.4649	−0.1667	−0.1629	0.047*	
H8B	0.4765	−0.1737	−0.0785	0.047*	
H8C	0.5424	−0.2542	−0.1067	0.047*	
C9	0.51410 (14)	0.0797 (2)	−0.10192 (12)	0.0381 (5)	
H9A	0.4705	0.0884	−0.1516	0.046*	
H9B	0.5530	0.1584	−0.0907	0.046*	
H9C	0.4846	0.0747	−0.0669	0.046*	
C10	0.61356 (14)	−0.0416 (2)	−0.15144 (11)	0.0380 (5)	
H10A	0.5701	−0.0439	−0.2014	0.046*	
H10B	0.6530	−0.1191	−0.1439	0.046*	
H10C	0.6472	0.0425	−0.1440	0.046*	

### Atomic displacement parameters (Å^2^)

**Table:**

	*U*^11^	*U*^22^	*U*^33^	*U*^12^	*U*^13^	*U*^23^
Cl1	0.0473 (3)	0.0452 (3)	0.0496 (3)	0.0194 (3)	0.0291 (3)	0.0033 (2)
P1	0.0271 (2)	0.0159 (2)	0.0265 (2)	−0.00014 (17)	0.01385 (19)	0.00075 (17)
O1	0.0427 (8)	0.0170 (7)	0.0628 (10)	0.0058 (6)	0.0291 (8)	0.0069 (6)
O2	0.0354 (7)	0.0170 (6)	0.0402 (8)	0.0001 (5)	0.0216 (6)	−0.0002 (5)
N1	0.0280 (8)	0.0140 (7)	0.0319 (8)	−0.0010 (6)	0.0157 (6)	0.0008 (6)
N2	0.0405 (9)	0.0180 (7)	0.0265 (8)	−0.0030 (7)	0.0122 (7)	−0.0004 (6)
N3	0.0278 (8)	0.0151 (7)	0.0313 (8)	−0.0003 (6)	0.0107 (7)	0.0012 (6)
C1	0.0246 (8)	0.0190 (9)	0.0266 (9)	0.0035 (7)	0.0090 (7)	0.0023 (7)
C2	0.0355 (10)	0.0263 (10)	0.0390 (11)	0.0072 (8)	0.0224 (9)	0.0028 (8)
C3	0.0292 (9)	0.0277 (10)	0.0274 (9)	0.0009 (8)	0.0106 (8)	−0.0018 (8)
C4	0.0477 (14)	0.0489 (14)	0.0491 (14)	−0.0168 (12)	0.0070 (11)	0.0031 (11)
C5	0.0546 (14)	0.0398 (12)	0.0302 (10)	0.0109 (11)	0.0115 (10)	0.0018 (9)
C6	0.0453 (13)	0.0766 (18)	0.0349 (12)	0.0185 (13)	0.0153 (10)	−0.0099 (12)
C7	0.0263 (9)	0.0257 (9)	0.0286 (9)	0.0006 (7)	0.0096 (7)	−0.0014 (7)
C8	0.0342 (11)	0.0327 (11)	0.0460 (12)	−0.0070 (9)	0.0092 (9)	−0.0028 (9)
C9	0.0351 (11)	0.0327 (11)	0.0420 (12)	0.0090 (9)	0.0086 (9)	0.0019 (9)
C10	0.0357 (11)	0.0500 (13)	0.0278 (10)	−0.0014 (10)	0.0110 (9)	−0.0052 (9)

### Geometric parameters (Å, °)

**Table:**

Cl1—C2	1.7647 (19)	C4—H4C	0.9800
P1—O2	1.4803 (13)	C5—H5A	0.9800
P1—N3	1.6241 (16)	C5—H5B	0.9800
P1—N2	1.6324 (16)	C5—H5C	0.9800
P1—N1	1.7030 (15)	C6—H6A	0.9800
O1—C1	1.214 (2)	C6—H6B	0.9800
N1—C1	1.350 (2)	C6—H6C	0.9800
N1—H1N	0.866 (9)	C7—C9	1.520 (3)
N2—C3	1.483 (2)	C7—C8	1.528 (3)
N2—H2N	0.850 (9)	C7—C10	1.533 (3)
N3—C7	1.486 (2)	C8—H8A	0.9800
N3—H3N	0.859 (9)	C8—H8B	0.9800
C1—C2	1.520 (2)	C8—H8C	0.9800
C2—H2A	0.9900	C9—H9A	0.9800
C2—H2B	0.9900	C9—H9B	0.9800
C3—C5	1.519 (3)	C9—H9C	0.9800
C3—C4	1.520 (3)	C10—H10A	0.9800
C3—C6	1.525 (3)	C10—H10B	0.9800
C4—H4A	0.9800	C10—H10C	0.9800
C4—H4B	0.9800		
			
O2—P1—N3	116.80 (8)	C3—C5—H5A	109.5
O2—P1—N2	115.32 (8)	C3—C5—H5B	109.5
N3—P1—N2	102.57 (8)	H5A—C5—H5B	109.5
O2—P1—N1	103.86 (7)	C3—C5—H5C	109.5
N3—P1—N1	109.50 (8)	H5A—C5—H5C	109.5
N2—P1—N1	108.65 (8)	H5B—C5—H5C	109.5
C1—N1—P1	124.72 (12)	C3—C6—H6A	109.5
C1—N1—H1N	116.1 (14)	C3—C6—H6B	109.5
P1—N1—H1N	119.1 (14)	H6A—C6—H6B	109.5
C3—N2—P1	126.36 (13)	C3—C6—H6C	109.5
C3—N2—H2N	118.8 (15)	H6A—C6—H6C	109.5
P1—N2—H2N	114.8 (15)	H6B—C6—H6C	109.5
C7—N3—P1	128.25 (12)	N3—C7—C9	110.09 (15)
C7—N3—H3N	114.5 (14)	N3—C7—C8	105.57 (15)
P1—N3—H3N	112.5 (14)	C9—C7—C8	109.82 (17)
O1—C1—N1	124.08 (17)	N3—C7—C10	111.48 (15)
O1—C1—C2	123.48 (16)	C9—C7—C10	109.86 (17)
N1—C1—C2	112.41 (15)	C8—C7—C10	109.94 (17)
C1—C2—Cl1	111.84 (13)	C7—C8—H8A	109.5
C1—C2—H2A	109.2	C7—C8—H8B	109.5
Cl1—C2—H2A	109.2	H8A—C8—H8B	109.5
C1—C2—H2B	109.2	C7—C8—H8C	109.5
Cl1—C2—H2B	109.2	H8A—C8—H8C	109.5
H2A—C2—H2B	107.9	H8B—C8—H8C	109.5
N2—C3—C5	107.42 (16)	C7—C9—H9A	109.5
N2—C3—C4	111.14 (17)	C7—C9—H9B	109.5
C5—C3—C4	108.87 (18)	H9A—C9—H9B	109.5
N2—C3—C6	109.32 (16)	C7—C9—H9C	109.5
C5—C3—C6	109.85 (18)	H9A—C9—H9C	109.5
C4—C3—C6	110.2 (2)	H9B—C9—H9C	109.5
C3—C4—H4A	109.5	C7—C10—H10A	109.5
C3—C4—H4B	109.5	C7—C10—H10B	109.5
H4A—C4—H4B	109.5	H10A—C10—H10B	109.5
C3—C4—H4C	109.5	C7—C10—H10C	109.5
H4A—C4—H4C	109.5	H10A—C10—H10C	109.5
H4B—C4—H4C	109.5	H10B—C10—H10C	109.5
			
O2—P1—N1—C1	−177.92 (15)	P1—N1—C1—C2	175.25 (13)
N3—P1—N1—C1	−52.46 (17)	O1—C1—C2—Cl1	−9.6 (3)
N2—P1—N1—C1	58.83 (17)	N1—C1—C2—Cl1	172.32 (13)
O2—P1—N2—C3	−29.69 (19)	P1—N2—C3—C5	−164.41 (15)
N3—P1—N2—C3	−157.76 (15)	P1—N2—C3—C4	−45.4 (2)
N1—P1—N2—C3	86.38 (16)	P1—N2—C3—C6	76.4 (2)
O2—P1—N3—C7	55.94 (17)	P1—N3—C7—C9	−56.2 (2)
N2—P1—N3—C7	−176.93 (15)	P1—N3—C7—C8	−174.63 (14)
N1—P1—N3—C7	−61.68 (16)	P1—N3—C7—C10	66.0 (2)
P1—N1—C1—O1	−2.8 (3)		

### Hydrogen-bond geometry (Å, °)

**Table:**

*D*—H···*A*	*D*—H	H···*A*	*D*···*A*	*D*—H···*A*
N1—H1N···O2^i^	0.87 (1)	1.92 (1)	2.778 (2)	175 (2)
N2—H2N···O1^ii^	0.85 (1)	2.56 (1)	3.337 (2)	153.(2)
N3—H3N···O1^ii^	0.86 (1)	2.12 (1)	2.979 (2)	175 (2)

Symmetry codes: (i) −*x*+3/2, −*y*+1/2, −*z*; (ii) −*x*+3/2, −*y*−1/2, −*z*.
